# Whole transcriptome data analysis of mouse embryonic hematopoietic stem and progenitor cells that lack Geminin expression

**DOI:** 10.1016/j.dib.2016.03.028

**Published:** 2016-03-12

**Authors:** Alexandra L. Patmanidi, Nikolaos I. Kanellakis, Dimitris Karamitros, Christos Papadimitriou, Zoi Lygerou, Stavros Taraviras

**Affiliations:** a Laboratory of Physiology, Medical School, University of Patras, Patras, Greece; b Laboratory of General Biology, Medical School, University of Patras, Patras, Greece

## Abstract

We performed cDNA microarrays (Affymetrix Mouse Gene 1.0 ST Chip) to analyze the transcriptome of hematopoietic stem and progenitor cells (HSPCs) from E15.5dpc wild type and Geminin (Gmnn) knockout embryos. Lineage negative cells from embryonic livers were isolated using fluorescence activated cell sorting. RNA samples were used to examine the transcriptional programs regulated by Geminin during embryonic hematopoiesis. The data sets were analyzed using the GeneSpring v12.5 platform (Agilent). The list of differentially expressed genes was filtered in meta-analyses to investigate the molecular basis of the phenotype observed in the knockout embryos, which exhibited defective hematopoiesis and death. The data from this study are related to the research article “Geminin deletion increases the number of fetal hematopoietic stem cells by affecting the expression of key transcription factors” (Karamitros et al., 2015) [1].

The microarray dataset has been deposited at the Gene Expression Omnibus (GEO) under accession GEO: GSE53056.

**Specifications Table**

**Table:** 

Subject area	Biology
More specific subject area	Developmental/Stem cell biology
Type of data	Figure (clustering), graph, table
How data was acquired	cDNA microarrays using GPL6246 [MoGene-1_0-st] Affymetrix Mouse Gene 1.0 ST Array [transcript (gene) version]. Organism: *Mus Musculus.* Strain background: C57BL6. Developmental stage: E15.5. Tissue: Fetal liver. Cell type: Lineage negative embryonic hematopoietic cells, Genotype: Geminin^wt/wt^ or Geminin ^fl/wt^ (control) [2] and Geminin^fl/ko^ Vav1:iCre [2, 3] (knock out) mouse lines
Data format	Analyzed using Genespring v12.5
Experimental features	Wild type and embryos lacking Geminin from the hematopoietic stem and progenitor cells (HSPCs); RNA from HSPCs; HSCPs were isolated using FACS sorting from dissected fetal livers
Data source location	Department of Physiology, Medical School, University of Patras, Greece
Data accessibility	The data within this article and is publicly available at the Gene Expression Omnibus (GEO) under accession GEO: GSE53056.

**Value of the data**

• Transcriptomics analysis of embryonic hematopoietic stem and progenitor cells that lack Geminin expression is described.
• The present dataset relates to defective hematopoiesis with a differentiation block early in the hematopoietic hierarchy. It is anticipated that similar phenotypes can be interpreted using comparative analyses with our data.
• Moreover, the list of differentially expressed genes includes changes in transcription factors and epigenetic regulators that can link Geminin, a cell cycle inhibitor, with novel cellular pathways.
• Finally, this dataset can serve as a reference point for various studies pertaining to hematopoietic cell lineage commitment/differentiation and hematopoietic malignancies.

## 1 Data

**Fig. 1:**
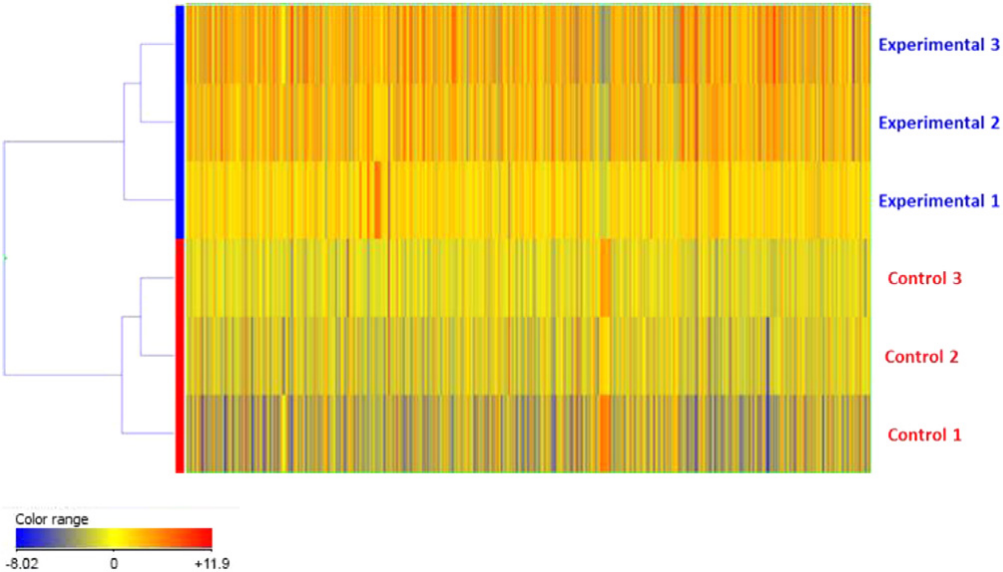
Qualitative evaluation of samples analyzed in the microarrays. Unsupervised hierarchical clustering: the heatmaps provide a qualitative evaluation of the three replicates of each group with regard to their gene expression profiles, the degree of heterogeneity among replicates, and their classification as control versus experimental. The heatmap legend indicates the fold change value range.

**Fig. 2:**
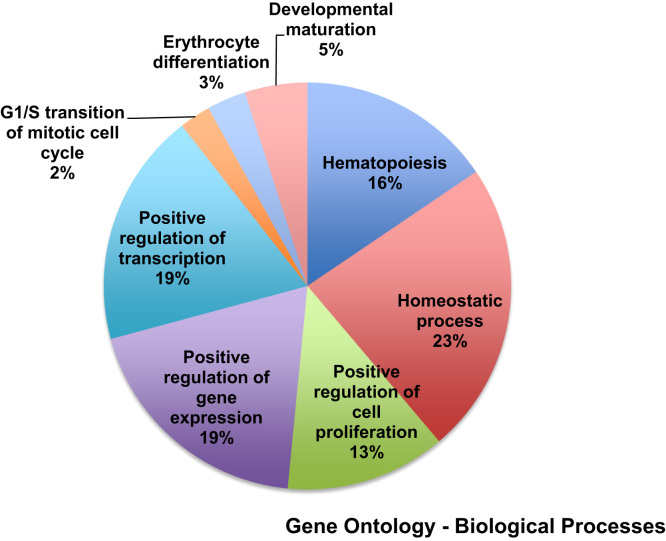
Gene ontology – Biological Processes. Among the top hits for biological processes identified by DAVID and GSEA were hematopoiesis, homeostasis, transcription and cell cycle regulation.

Here we present a qualitative evaluation of microarray data in the format of heatmaps, following hierarchical clustering analysis (Fig. 1). Additionally, we have included a concise set of meta-analyses, both Gene Ontology using DAVID and GSEA (Fig. 2), as well as a listing of the KEGG pathways (Table 1) affected by the differentially expressed genes [4], [5], [6]. Briefly, the dataset is from whole transcriptome analysis of hematopoietic stem and progenitor cells (HSPCs) isolated from E15.5dpc embryo livers. In this study, control and Geminin knockout littermate embryos were utilized in order to examine the role of Geminin in the developing mouse hematopoietic system. Control were those embryos whose genotype had both Geminin alleles intact, whereas both alleles were disrupted/absent in the Geminin^fl/ko^ Vav1:iCre counterparts in a tissue-specific manner [1], [2].

The transcriptome of both groups was examined by means of cDNA microarrays and differentially expressed genes are presented as fold change of knockout versus control expression levels.

Direct link to deposited files.

http://www.ncbi.nlm.nih.gov/geo/query/acc.cgi?acc=GSE53056

## 2 Experimental design, materials and methods

### 2.1 Sample preparation

Lineage negative hematopoietic stem and progenitor cells from homogenized fetal livers from E15.5 embryos were isolated by FACS sorting with known blood cell lineage markers (biotinylated CD3, CD8, CD19, B220, Ter119, CD11B, CD11C, GR1; all antibodies were from eBioscience and detection was with APC-conjugated streptavidin).

### 2.2 RNA extraction

Total RNA was extracted from FACS-sorted cells using the RNeasy MicroKit (Qiagen). RNA integrity was assessed with an Agilent 2000 Bioanalyzer.

### 2.3 Microarray preparation

Samples were enzymatically fragmented and biotinylated using the WT Terminal Labeling Kit (Affymetrix). Samples were hybridized using Affymetrix hybridization kit materials. Washing was done in GeneChip Fluidics Station 450. The chips were scanned using the Affymetrix Scanner 3000 7G with autoloader.

### 2.4 Microarray data analysis

The data were processed and analyzed using GeneSpring (v.12.5, Agilent). Statistical significance was assessed using Student׳s *T*-test unpaired (two separate sets of independent and identically distributed samples) with a 95% confidence interval (*P*-value<0.05). The cutoff value for the fold change of the genes was set to be greater than 1.5. The analysis was carried out using the genotype criterion, but non-averaged for the three biological replicates.

## Figures and Tables

**Table 1:** KEGG Pathways.

**Term**	**Gene count**	***P*-value**
mmu04630:Jak-STAT signaling pathway	31	9.41E-05
mmu04070:Phosphatidylinositol signaling system	19	1.98E-04
mmu04370:VEGF signaling pathway	18	7.13E-04
mmu04010:MAPK signaling pathway	43	7.22E-04
mmu04640:Hematopoietic cell lineage	19	8.63E-04
mmu04062:Chemokine signaling pathway	31	0.002207624
mmu05221:Acute myeloid leukemia	14	0.002453712
mmu05340:Primary immunodeficiency	10	0.005878255
mmu04310:Wnt signaling pathway	24	0.015019777
mmu05200:Pathways in cancer	43	0.025504562
mmu04620:Toll-like receptor signaling pathway	17	0.02629612
mmu04020:Calcium signaling pathway	27	0.04331303
